# Assessment of *Alternaria* Toxins and Pesticides in Organic and Conventional Tomato Products: Insights into Contamination Patterns and Food Safety Implications

**DOI:** 10.3390/toxins17010012

**Published:** 2024-12-29

**Authors:** Tommaso Pacini, Teresa D’Amore, Stefano Sdogati, Emanuela Verdini, Rita Bibi, Angela Caporali, Elisa Cristofani, Carmen Maresca, Serenella Orsini, Alessandro Pelliccia, Eleonora Scoccia, Ivan Pecorelli

**Affiliations:** 1 Istituto Zooprofilattico Sperimentale dell’Umbria e delle Marche “Togo Rosati”, 06126 Perugia, Italy; t.pacini@izsum.it (T.P.); e.verdini@izsum.it (E.V.); r.bibi@izsum.it (R.B.); a.caporali@izsum.it (A.C.); e.cristofani@izsum.it (E.C.); c.maresca@izsum.it (C.M.); s.orsini@izsum.it (S.O.); a.pelliccia@izsum.it (A.P.); e.scoccia@izsum.it (E.S.); i.pecorelli@izsum.it (I.P.); 2 Laboratory of Preclinical and Translational Research, IRCCS CROB, Centro di Riferimento Oncologico della Basilicata, 85028 Rionero in Vulture, Italy

**Keywords:** *Alternaria* toxins, pesticides, tomato sauces, mass spectrometry, analytical controls, risk assessment, genotoxic and carcinogenic products, organic vs. conventional crops, pest and mycotoxin management measures

## Abstract

*Alternaria* toxins (ATs) are a group of toxins produced by *Alternaria* fungi that frequently contaminate tomatoes and tomato products. Recently, the European Food Safety Authority evaluated ATs for their genotoxic and carcinogenic properties. *Alternaria* infestation is often controlled using ad hoc treatment strategies (fungicides). In this study, two analytical methods were developed, validated and applied for the determination of five ATs and 195 pesticides in tomato products collected from the Italian market. Two distinct groups, organic (n = 20) and conventional (n = 20) Italian tomato sauces, were characterized in depth. Tenuazonic acid, alternariol and alternariol monomethyl ether were found up to 517, 27 and 7.1 µg/kg, respectively, while pesticides were detected between 0.0026 and 0.0421 mg/kg in conventional products, and, interestingly, up to 0.0130 mg/kg in organic products. No correlation emerged between the detected levels of ATs and pesticides and the type of tomato cultivation, but the probability of pesticide contamination in conventional products was eight times higher than in organics. Some considerations about exposure assessment and risk characterization for ATs were also proposed in the overall population and in more sensitive and/or exposed subgroups, underlining the need for new focused toxicological and monitoring studies to establish reliable reference values. Moreover, these data highlight that fungicide treatments may not protect tomatoes from ATs contamination, although it may remove fungi infestation. As organic product consumption is increasing, it is important to lay down dedicated regulations for maximum permitted levels to ensure the food safety of these products that are often perceived by consumers as a healthier and environmentally friendlier choice.

## 1. Introduction

Tomato (*Solanum lycopersicum*), a key component of the Mediterranean diet and one of the most consumed vegetables worldwide, is rich in antioxidants such as lycopene, β-carotene, vitamin C, and phenolic compounds, which support longevity and reduce the risk of chronic disease [1]. China contributes approximately 35% to the global tomato production while in Europe, Italy and Spain lead the market, producing around 6.5 million and 4.4 million tons, respectively. A significant portion of this yield is processed into various tomato-based products (tomato sauce, tomato puree, etc.) [2]. Tomatoes are highly perishable fruits due to their soft epidermis that offers low resistance to environmental damages that may occur during cultivation, transportation and storage. Fungal infestation is one of the causes of tomato rotting, especially before harvesting, with the *Alternaria* genus as the most common fungi to contaminate these products [3]. *Alternaria* spp. can produce up to 70 toxic secondary metabolites, with several compounds exhibiting cytotoxic, genotoxic, teratogenic and potentially carcinogenic properties [4]. In 2011, the European Food Safety Authority (EFSA) published the first report on the occurrence of *Alternaria* toxins (ATs) in food and feed, reporting that certain ATs, especially alternariol (AOH) and alternariol-monomethyl ether (AME), may be considered compounds of high concern due to several toxicological evidences of DNA-damaging, intercalating and cross-linking activity [5]. Five years later, the EFSA assessed the population exposure, concluding that infants and toddlers were more exposed to tenuazonic acid (TeA) and AME, while vegetarians were more exposed to tentoxin (TEN) [6]. Other relevant ATs considered by both the EFSA evaluations were altenuene (ALT), altertoxins (ATX-I, II and III) and *A. alternata* f. sp. *Lycopersici* toxins (AAL TA1, TA2, TB1 and TB2) [4]. In 2022, the European Commission (EC) published a recommendation establishing indicative levels for TeA, AME and AOH in cereals, tomato products, spices, oilseeds and baby food [7]. *Alternaria* infestation manifests in characteristic symptoms on fruits and leaves, known as early blight (EB) and brown spots (BS) caused by *A. solani* and *A. alternata*, respectively [8]. Risk management measures include agronomic and physical controlling methods such as resistant cultivars, field drip irrigation and removing infected tomato leaves. Where these methods fail, fungicides, a well-known class of pesticides, are a feasible option with azoxystrobin, boscalid, cymoxanil, mancozeb, maneb, methiram and tebuconazole being common choices; though, more eco-friendly strategies are under investigation [9,10,11,12]. In the European Union (EU), maximum residue levels (MRLs) of pesticides are established by Regulation (EC) No 396/2005, which specifies permissible limits for each substance in different commodities [13]. Regulation (EU) No 1165/2021 governs organic farming, largely excluding pesticides except for spinosad and lambda-cyalothrin/deltamethrin insect traps [14]. Accidental contamination of organic products may occur, and the International Federation of Organic Agriculture Movements (IFOAM) proposed a tolerance guideline in 2012, setting a practical action level at 0.010 mg/kg based on Regulation (EU) No 609/2013 for infant and on-follow formula [15,16].

In this complex scenario, the importance of analytical controls and monitoring activities by official laboratories is increasing alongside the need for highly sensitive, multi-analyte methods for accurate multi-class determination of contaminants and pollutants [17]. For nearly all classes of mycotoxins and a broader range of pesticide residues (including ionic and polar compounds), liquid chromatography coupled with mass spectrometry (LC-MS) and high-resolution mass spectrometry (LC-HRMS) have become the preferred analytical approaches. These techniques provide advanced solutions for detecting both pesticides and mycotoxins across manifold matrices. Recent improvements, including enhanced software tools, comprehensive databases, and reduced reliance on analytical standards in screening protocols, are expected to further expand their application [18,19,20,21]. The EU lays down multi-annual controlling plans (MACP) for up to 250 pesticides and mixtures in different foods, highlighting the need for multiresidue methodologies capable of detecting hundreds of compounds in a single analysis [22,23]. For certain well-established classes of high-volatility pesticides, such as organochlorines and pyrethroids, detection via mass spectrometry is often preceded by gas chromatography (GC) separation. Advances in fast GC and two-dimensional GC (GC × GC-MS) have further enhanced GC-MS utility in food safety by providing rapid, high-resolution analysis. However, GC is rarely used in mycotoxin analysis, as mycotoxins are typically polar, non-volatile compounds that often require derivatization for detection [24,25,26]. Although fewer mycotoxins are regulated compared to pesticides, the multiresidue approach is increasingly applied in mycotoxin monitoring, especially for key toxins such as aflatoxins, fumonisins, T-2 and HT-2 toxins, ochratoxins, zearalenone and deoxynivalenol [27,28,29,30]. ATs are considered emerging mycotoxins and are often determined through single-class methods in products such as cereals, fruits and vegetables [31].

Consequently, in this study, we aimed to assess the occurrence and characterize the risk associated with ATs and pesticide residues in organic and conventional tomato products from the Italian market and identify potential correlations between the presence or absence of these substances and the type of product analyzed. We analyzed forty tomato sauce samples (twenty organic and twenty conventional) for five ATs (ALT, AME, AOH, TeA and TEN) using a triple-quadrupole mass spectrometer (LC-MS/MS) and screened for 195 pesticides using a LC-HRMS platform. Developing sensitive and reliable LC-MS methods is essential to reduce left-censored data in exposure assessments, and the data obtained will provide insights into contamination levels and prevalence, especially in organic products, which are increasingly consumed worldwide.

## 2. Results

### 2.1. Method Validation

Both methods were developed in accordance with the most up-to-date guidelines and validated in terms of specificity, linearity, limit of quantitation (LOQ), limit of detection (LOD), accuracy, expressed as the sum of trueness (recovery percentage) and precision under repeatability conditions (RSD_r_) [32,33,34,35]. Specificity criteria were evaluated by comparing analyte RTs and ion ratio values in incurred/spiked samples with the ones obtained by standard solutions, given a tolerance window of ±0.1 min and ±30%, respectively [33]. Linearity was compliant for each point of the calibration curves with deviations from true concentrations always ≤20%. Practical LOQs complied with Regulation (EU) No 2782/2023, reporting that LOQs should be at least half of the relevant maximum limit (ML) permitted for a substance [36]. For pesticide analysis, most target analytes showed a calculated LOD of 0.0008 mg/kg and an experimental LOQ of 0.0025 mg/kg. Recoveries were from 89 to 112% for ATs and from 60 to 133% for pesticides with the RSD_r_ always lower than 9.9 and 25% for ATs and pesticides, respectively. In detail, for pesticides, 72% of the analytes (group 1) showed a LOQ of 0.0025 mg/kg, while the other 27% showed a LOQ of 0.0050 mg/kg (group 2), and only the remaining 1% were higher (LOQ of 0.0100 mg/kg—group 3). Of 195 total analytes, 71% showed a recovery percentage between 90 and 110% at low concentrations (0.0025 mg/kg) while 81% of them were in the same range at high concentrations (0.010 mg/kg). The lowest recovery rate was observed for fenbutatin oxide (60% for both low and high concentrations) and the highest for bitertanol at low concentrations (133% at 0.0025 mg/kg) and for triadimenol at high concentrations (120% at 0.0100 mg/kg). Relevant validation parameters for both ATs and pesticides are summarized in Table 1. Comprehensive details on method development, standards, and validation parameters are provided in Tables S1–S5 and elaborated in the Materials and Methods section below.

### 2.2. Occurrence of ATs and Pesticides

Four (AME, AOH, TeA and TEN) out of five monitored ATs were found at >LOQ levels in both organic (O) and conventional (C) tomato sauces with similar incidences of >LOQ samples (O: 65%; C: 70%). Among these, TeA was found at the highest concentration in the ranges 75–379 µg/kg and 75–517 µg/kg in organic and conventional products, respectively. AOH was found in the ranges 1.5–18 µg/kg (O) and 1.6–27 µg/kg (C). AME was detected, on average, at lower concentrations, between 3.0–5.6 µg/kg in organic products and 2.2–7.1 µg/kg in their conventional counterparts. Interestingly, TEN was found only once (1.6 µg/kg in an organic tomato sauce) while ALT was never detected. MRLs are not set for ATs, although indicative levels were reported in Recommendation (EC) 553/2022; in tomato products these levels are set at 500 µg/kg, 10 µg/kg and 5 µg/kg for TeA, AOH and AME, respectively [7]. Among the analyzed samples, three out of forty (7.5%) were above the indicative levels laid down by the EU.

**Figure 1:**
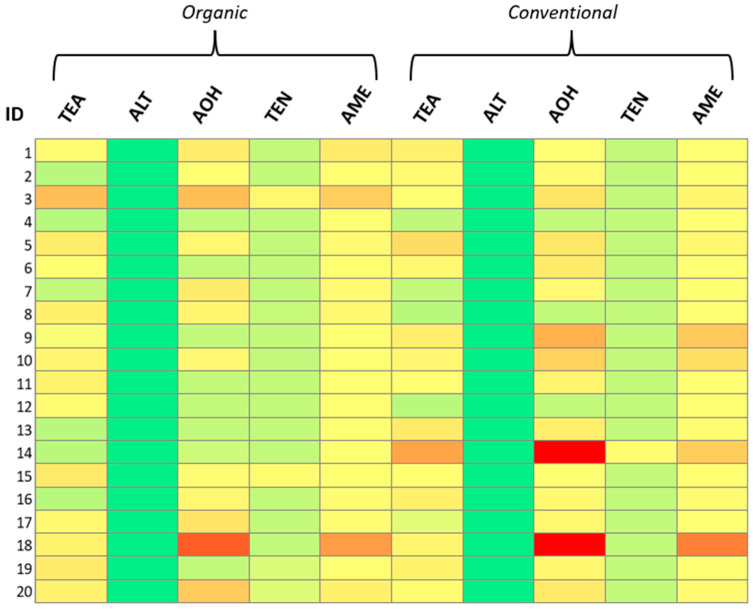
Heatmap of ATs contamination. Color range is set for each analyte from green (<LOD) to red (maximum detected value). LOD: 23 µg/kg (TeA); 0.5 µg/kg (other ATs). Maximum detected values: 517 µg/kg (TeA); 27 µg/kg (other ATs).

**Figure 2:**
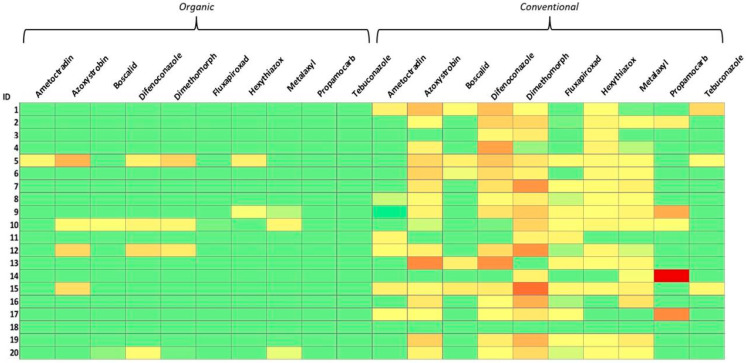
Heatmap of pesticide contamination. Color range is set for each analyte from green (<LOD) to red (maximum detected value). LOD: 0.0008 mg/kg. Maximum detected value: 0.0421 mg/kg.

For pesticides, 23 out of 40 (58%), were contaminated with ten different pesticides (ametoctradin, azoxystrobin, boscalid, difenoconazole, dimethomorph, fluxapyroxad, hexythiazox, metalaxyl, propamocarb and tebuconazole) with concentrations higher than LOQ. In addition, two organic samples showed levels of contamination between LOD and LOQ for hexythiazox and metalaxyl (O9) and for boscalid, difenoconazole, dimetomorph and metalaxyl (O20). Among the positive samples, the highest pesticide level was reported for sample C14 (propamocarb: 0.0421 mg/kg); this value is sensibly lower than the relevant MRL for this compound in tomatoes (2 mg/kg). The total incidence of pesticides detected on biological products >LOD was 30%. For the quantified pesticides >LOQ, this factor decreased to 20%. In conventional tomato sauces the scenery appears completely different with 95% of the samples testing positive to several pesticides in every case >LOQ. Considering the total amount of analyzed samples, 63% of them contained pesticides: dimethomorph was the most detected with 22 samples out of 25 (88%) having a concentration ranging from 0.0031 mg/kg (C6) to 0.0247 mg/kg (C15). This was immediately followed by difenoconazole, with 21 samples out of 25 (84%), and azoxystrobin, with 20 samples out of 25 (80%). These ranged from a minimum of 0.0026 mg/kg (O10) to a maximum of 0.0188 mg/kg (C13), 0.0027 mg/kg (C20) and 0.0203 mg/kg (C13), respectively. Among the organic samples, 14 out of 20 did not show any presence of pesticides (70%) whereas there was only one (5%) among the conventional products. The kind of pesticide residues found and quantified in the organic tomato sauces (4 out of 20) did not show significative differences compared to conventional products, even if the concentrations were generally lower, with difenoconazole, dimethomorph and azoxystrobin quantified in three cases out of four in ranges from 0.0026 (O10) to 0.0068 mg/kg (O12), 0.0044 mg/kg (O12) to 0.0085 mg/kg (O5) and 0.0067 (O15) to 0.0134 mg/kg (O5), respectively. Statistical qualitative and quantitative evaluation of the obtained data was carried out to determine correlations between the type of tomato cultivation and the levels of ATs and pesticides. Briefly, a descriptive data analysis was performed between organic and conventional products using a Pearson chi-squared test. Quantitative data were evaluated using a non-parametric Wilcoxon–Mann–Whitney test. The threshold for significance was set, in both cases, at *p*-value < 0.05. ATs contamination (expressed as a percentage of samples above LOQ) was not correlated to the type of product (*p*-value = 0.62); the same outcome was observed for the average concentrations of TeA (*p*-value: 0.91), AOH (*p*-value: 0.61) and AME (*p*-value: 1.00). On the contrary, conventional tomato sauces exhibited eight-times higher probability of pesticide contamination >LOQ with respect to organics (OR: 8.05; IC95%: 3.85–16.82). Differences between AT and pesticide contamination patterns are visualized in Figure 1 and Figure 2.

Regarding the concentrations of individual pesticides, while the maximum detected levels of certain pesticides differ between organic and conventional tomato sauces, organic products did not exhibit statistically significant differences in their overall contamination profile compared to conventional products. In other words, any organic sample tested positive aligns closely with the average pesticide concentration and variety profile found in the conventional samples.

## 3. Discussion

### 3.1. Occurrence of Alternaria Toxins and Pesticides in Tomato Products: General Considerations and Comparison with Previous Studies

In recent years, a substantial body of scientific literature has been focusing on the occurrence of ATs in food, with particular attention given to tomato products due to their high global consumption. Table 2 provides a summary of recent relevant studies, highlighting AT contamination levels in tomato sauces. TeA emerged as the most frequently detected AT with prevalence above LOQ in approximately 95% of the samples. Contamination levels were generally higher than the other ATs in tomato sauces (up to 485 µg/kg); occasionally, in tomato concentrates, TeA levels rose up to 5955 µg/kg [37]. AOH and AME were other commonly detected toxins in tomato products with average levels much lower than TeA. Other ATs such as TEN, ALT, altertoxin and altenusin were infrequently reported. Data obtained by our investigations align with the existing literature, particularly in terms of the average contamination and prevalence of certain toxins.

Phytosanitary products are often applied to achieve high crop yields, with bactericides and fungicides frequently used preventively even before visible plant disease symptoms. However, *Alternaria* infestation may precede these treatments, resulting in toxin contamination in the final product despite the preventive application. Only a few studies report relevant pesticide levels in processed tomato products. Abd-Elhaleem et al. analyzed five tomato paste samples reporting boscalid and carbendazim up to 0.03 and 0.01 mg/kg, respectively [42]. In 2022, Balkan et al. applied a multiresidue method to analyze 20 tomato sauces reporting pesticide levels between 0.010 and 0.094 mg/kg, including acetamiprid, ametoctradin, metalaxyl and imazalil sulfate [43]. Our data are consistent with these studies and highlight that nearly every conventional sample analyzed contained at least one pesticide above LOQ (0.0025 mg/kg) with levels ranging from 0.0025 to 0.0421 mg/kg. Even though incidence is high, it is notable to see that these levels are widely lower than MRLs.

A separate evaluation must be carried out for organic products. Currently, Regulation (EC) No 396/2005 does not establish pesticide MRLs for these specific products which follow a separate legislation based on Regulation (EC) No 609/2013 setting practical action levels at 0.010 mg/kg. Despite pesticide use being prohibited in organic farming, approximately 20% of organic samples had pesticide levels above the LOQ [14]. In one case (sample B5), azoxystrobin levels exceeded the practical action level and additional pesticides were also detected, including difenoconazole (0.0034 mg/kg), dimethomorph (0.0085 mg/kg) and hexythiazox (0.0035 mg/kg). Although the case is particularly interesting, not enough information is available to suggest a possible illegal treatment of organic products. Accidental contamination using polluted irrigation water cannot be excluded. In this regard, in a report analyzing pesticide detection in surface waters, rivers and groundwaters from 2016 to 2021, the European Environment Agency (EEA) highlighted that in many countries, up to 50% of water basins contained pesticides exceeding the precautionary threshold of 0.1 µg/L set by Directive 2000/60/EC [44,45]. A limit of 0.01 mg/kg for unintended pesticides in organic food was established in Italy by the Ministerial Decree (MD) of 13 January 2011 (published in the *Official Italian Gazette*) above which products cannot be marketed as organic, whatever the cause of the contamination is.

The contamination profile of some samples is especially interesting in light of recent regulatory actions and upcoming reviews of certain pesticide approvals. In addition to the non-renewal of dimethomorph in 2024 due to concerns over its endocrine-disrupting properties and reproductive toxicity, the approvals for azoxystrobin and difenoconazole are set to expire in 2024 and 2026, respectively [46]. These substances are widely used in tomato farming for their effectiveness against fungal pathogens. However, their pending expiration underlines a pressing need for alternative methods to maintain crop health and quality, especially as the regulatory environment moves toward stricter safety standards.

The non-renewal of dimethomorph also raises questions regarding the effectiveness of pesticide treatments in reducing mycotoxin contamination, particularly for ATs. Despite dimethomorph application, this study reveals no significant reduction in ATs in conventional tomato products, suggesting that while fungicides may limit fungal growth, they do not necessarily prevent mycotoxin formation. This supports broader findings that mycotoxins can persist even after pesticide treatment, pointing to a need for alternative approaches to control fungal contamination. The potential withdrawal of these fungicides from the market presents both challenges and opportunities. Conventional farming systems may need to adapt quickly, integrating more sustainable practices such as advanced integrated pest management and exploring non-chemical methods [47]. For organic production, where occasional contamination with these pesticides is observed, there is an added incentive to enhance preventive measures and monitoring studies to avoid cross-contamination from surrounding environments.

### 3.2. Considerations on Dietary Exposure and Risk Characterization

The data obtained from this study, though based on a limited number of samples, can be used for some risk assessment considerations, particularly concerning ATs. In fact, thanks to the low levels of left-censored data, except for TEN, the data are particularly interesting (Table S6). These contaminants represent an emerging risk for which insufficient toxicological data have so far prevented the establishment of reliable reference values. Consequently, the Threshold of Toxicological Concern (TTC) is employed as a risk assessment tool to define safe exposure levels for ATs and other chemicals when specific toxicological data are lacking. The TTC approach applies a conservative threshold based on chemical structure, anticipated toxicity and exposure level; thus, providing a quantitative framework for assessing compounds with limited or no direct data. This method is valuable in the initial stages of safety assessment for food contaminants, facilitating regulatory decisions regarding acceptable exposure limits [48,49]. In this context, a TTC of 2.5 ng/kg body weight per day (bw day) has been set for AOH and AME, and 1500 ng/kg bw day for TeA and TEN. For AOH and AME, this conservative threshold is warranted due to their cytotoxic, genotoxic and potentially carcinogenic properties. In fact, AOH and AME have been shown to inhibit topoisomerase enzymes, which can result in DNA damage and may contribute to mutagenic and carcinogenic effects [50].

Based on the mean concentrations of ATs detected in this study, a potential exposure scenario was proposed for global, European and Italian populations. Dietary exposure was estimated using tomato product consumption data (sauces and puree) from three major databases (FAO-WHO, Food-Ex and INRAN-SCAI). For the Italian population, various age groups were included: infants and toddlers (0–35 months), children (3–5 years), adolescents (6–14 years), young adults (15–49 years), older adults (50–74 years) and the elderly (≥75 years). High consumers (95th percentile, P-95)—individuals with particularly high tomato product intake—were also considered [51]. A summary of the findings is presented in Table S6.

The dietary exposure levels of the study (DELs) were calculated by combining the mean concentration of each AT with the consumption data and then compared against the established TTC. Generally, DELs were low; however, for the genotoxic compounds AOH and AME, exposure may exceed recommended thresholds, particularly within the Italian population and certain age groups. Among individuals aged 0–14 years, infants and toddlers showed higher exposure levels, likely due to less dietary variety. When comparing organic and conventional samples, the DELs for conventional samples were paradoxically slightly higher, though the difference was not statistically significant.

These data seem to be consistent with other studies [6,50], although the relatively small number of samples limits the potency of considerations. A key strength of this study is its alignment with EFSA recommendations to develop more sensitive analytical methods that reduce uncertainty in exposure assessments for various ATs, particularly by addressing the high proportion of left-censored data [5,6].

Another important aspect of this study is the investigation of the co-occurrence of multiple toxins, which may pose an additional risk to consumers, particularly within vulnerable subgroups of the population. The simultaneous presence of xenobiotics in food can lead to significant additive or synergistic effects compared to exposure to individual substances. For instance, a synergistic effect was observed in Caco-2 cells exposed to a 1:1 combination of AOH and AME (concentration range: 3.125–30 µM), where the mixture significantly reduced cell proliferation more than either AOH or AME alone [52,53].

Furthermore, this study is the first to highlight the co-occurrence of ATs and pesticides, which are compounds with complex modes of action that may collectively increase the risk for consumers. Rizzati et al., for example, demonstrated that pesticides interact at various cellular levels, complicating the prediction of their combined effects in mixtures [54]. Only a few authors tried to evaluate mycotoxin–pesticide mixtures on cells. Eze et al. suggested that organochlorine pesticides enhance deoxynivalenol, ochratoxin A and zearalenone toxicity in Leydig cells; in some cases, estrogen nuclear receptor activation potentially leading to adverse effects in male reproductivity was described [55,56]. Antagonistic mechanisms were reported by Tadee et al. involving aflatoxin B1 and chlorpyrifos and their action on HepG2 cells. Authors indicated a combination index of 1.67, excluding the additive or synergistic effects [57]. This highlights the need for more comprehensive assessments to understand the potential health impacts of such co-exposures [58,59].

## 4. Conclusions

This study provides novel insights into the occurrence of *Alternaria* toxins and pesticide residues in organic and conventional tomato products, highlighting important, previously unexplored aspects of food safety in these products. One key finding is that pesticide treatments, while effective in eliminating the fungus itself, may not prevent the occurrence of *Alternaria* toxins in tomatoes. This suggests that current mycotoxin and pest risk management practices might not adequately address contamination from some mycotoxins, especially for consumers who perceive organic products as inherently safe and “green”. Importantly, despite being limited to a restricted sample population, this study found no statistical correlation between *Alternaria* toxin contamination and cultivation type, challenging assumptions about the safety of organic versus conventional products. The detection of pesticides in 20% of organic tomato sauces, although at concentrations typically below 0.01 mg/kg, reveals a comparable contamination profile to conventional products. This pattern, alongside the absence of pesticides specifically allowed in organic production, suggests that contamination in organic sauces may derive from cross-contamination rather than direct application. For conventional farming, the relatively high levels of pesticide residues emphasize the need for integrated pest management strategies that can help reduce reliance on chemical pesticides. For organic products, the occasional detection of pesticides at elevated levels highlights a need for more rigorous compliance with organic standards and enhanced monitoring and controls to ensure product integrity.

The results indicate the need for more sensitive and targeted approaches in food safety and risk assessment, especially regarding *Alternaria* toxins. Given the limited toxicological data, focused toxicological studies are essential to derive reference values, enabling better risk assessment frameworks. In addition, the study highlights a critical gap in current fungal contamination management practices. The reliance on visual detection of blight spots to initiate fungicide application is probably insufficient for preventing mycotoxin contamination. There is an urgent need to develop and implement early detection methods for *Alternaria*. Such methods should include advanced molecular techniques or biosensors capable of identifying fungal presence before significant mycotoxin production occurs. The integration of early detection methods and more robust strategies could further safeguard consumer health, reduce exposure risks, and enhance the overall quality of tomato-based products.

In conclusion, this study provides valuable data on pesticide and *Alternaria* toxin contamination in tomato products, pointing to the need for improved toxicological studies, management strategies and regulatory standards across both organic and conventional food production. Implementing such measures would not only safeguard consumer health but also enhance the overall quality and safety of tomato-based food products.

## 5. Materials and Methods

### 5.1. Sample Collection

Between January and February 2024, forty tomato sauce samples were purchased from local markets (Umbria region, Italy), selecting twenty products labeled as “organic” (O) and twenty as non-organic, subsequently referred to as “conventional” (C). Only sauces made from Italian tomatoes were included. Collected samples were homogenized using a Retsch Grindomix GM300 (Haan, Germany) and stored at −20 °C until their analysis.

### 5.2. Standards and Reagents

ATs (ALT, AME, AOH, TeA and TEN) were purchased from Biopure (Romer Labs, Tulln, Austria). Isotopically labeled internal standards (ILISs) of ATs (ALT d_6_, AME d_3_, AOH d_3_, TeA ^13^C_2_ and TEN d_3_) were purchased from ASCA GmbH (Berlin, Germany). Strata XL SPE columns (200 mg/6 mL—Part No 8B-S043-FCH) were supplied by Phenomenex (Torrance, CA, USA). Tween 20 and ammonium acetate (LC-MS grade) were obtained from Sigma–Aldrich (St. Louis, MO, USA). Ammonium hydroxide (NH_4_OH) and acetic acid (AcOH) were purchased from Honeywell (Seelze, Germany). Ethyl acetate (EtOAc), methanol (MeOH) and acetonitrile (ACN) were obtained from Carlo Erba Reagents Srl (Milan, Italy). Pesticides were purchased as CRMs (Certified Reference Materials) from Lab Instruments (Castellana Grotte, Italy) as ACN solutions at 100 µg/mL. The CRMs were appropriately diluted to obtain a working solution mixture (1 µg/mL) that was used for matrix-matched calibration curves. All the purchased standards for pesticides are shown in Table S1. All the solvents used were LC-MS grade while the water was purified through a Millipore Milli-Q System (Merck KGaA, Darmstadt, Germany).

### 5.3. Sample Preparation

#### 5.3.1. Alternaria Toxins

Sample preparation was performed as reported by Lattanzio et al. [32]. Briefly, 2 g of tomato sauce were weighed in a 50 mL polypropylene tube, spiked with ATs ILIS solution (TeA^13^C_2_: 250 µg/kg; ALT d_6_, AME d_3_, AOH d_3_ and TEN d_3_: 5 µg/kg) and vortexed. The samples were extracted using 14 mL of a MeOH:H_2_O:AcOH 85:14:1 (*v*/*v*/*v*) solution and mixed using a horizontal shaker for 45′. Samples were centrifuged (22 °C, 2773 RCF, 15′) and 7.5 mL of the obtained supernatant was diluted with the same amount of AcOH 1%. The Strata XL SPE columns were conditioned with 7 mL of MeOH, H_2_O and AcOH 1% prior to the sample loading. The SPE columns were washed with 4 mL of AcOH 1% and the analytes were eluted with 7 mL of the MeOH:EtOAc 75:25 (*v*/*v*) mixture. Eluates were dried under a gentle nitrogen stream (50 °C) and reconstituted with 1 mL of a 1 mM ammonium acetate:MeOH mixture 75:25 (*v*/*v*) prior to the LC-MS/MS analysis.

#### 5.3.2. Pesticides

Extraction and purification for the determination of pesticides were performed following the European Standard method for the analysis of pesticide residues in foods of plant origin [33]. The tomato sauce samples were homogenized and then extracted with 10 mL of ACN in accordance with the Extraction module E1 reported in European Standard EN 15662:2018. In the same way, the salting out and purification were performed using the dispersive SPE–Modular QuEChERS method and Clean-up module C1. The extract was filtered with a PTFE filter and stabilized before an injection with a 5% solution of formic acid in acetonitrile as described in the same document (Module S1) [34]. The samples were directly injected into the UHPLC-HRMS system. Two positive controls at the concentrations of 0.0025 mg/kg and 0.0100 mg/kg were prepared for each analysis, and matrix-matched standards for linearity evaluation were prepared at five different levels (0.0025, 0.0050, 0.0100, 0.0250 and 0.0500 mg/kg).

### 5.4. LC-MS and LC-HRMS Methods

ATs and their corresponding labeled internal standards were determined with an UHPLC Shimadzu Nexera X2 (Kyoto, Japan) coupled with an AB Sciex Qtrap 6500+ (Framingham, MA, USA) mass spectrometer operating in negative ESI mode (ESI-). Analyte separation was obtained using an Ascentis Express C18 column (100 × 2.1 mm; 2.7 µm) supplied by Supelco (Bellefonte, PA, USA) with a 5 mM ammonium acetate buffer (pH = 8.0) and MeOH as mobile phases. The chromatographic gradient is detailed in the study by Lattanzio et al. [32] and optimized MS parameters are reported in Table S2.

Pesticides were analyzed through a LC-HRMS system purchased from AB Sciex. The instrument is composed of an UHPLC (Exion LC, AB Sciex, Framingham, MA, USA), a binary pump, a degassing unit and an autosampler coupled with a high-resolution quadrupole time-of-flight hybrid detector (UHPLC-HRMS Q-TOF) AB Sciex 6600+ (Framingham, MA, USA). The interface used for the ionization of the analytes was an electrospray operating in positive mode (ESI+). This HRMS instrument performs analysis in a mass/charge range from 5 to 2250 *m*/*z* with a resolution ≥ 30,000 FWHM at 813 *m*/*z* and ≥25,000 FWHM at 195 *m*/*z* with an accumulation time of 10 ms. Chromatographic and MS parameters together with the accurate mass of the precursor and fragment ions are shown in Table S3.

### 5.5. Validation Study

The ATs and pesticides method validations were carried out to assess specificity, linearity, LOD and LOQ, accuracy, expressed as the sum of trueness (recovery) and precision under repeatability conditions (RSD_r_). Specificity was evaluated in terms of analyte retention times (RTs) and ion ratio tolerance as detailed by document SANTE/11312/2021 v2 [33]. Linearity was assessed as back-calculated concentrations (BCC) of each point included in the calibration curves and the corresponding deviations from the true concentrations. The analytical linear range was 0–1000 ng/mL for TeA, 0–20 ng/mL for ALT, AME, AOH and TEN and 0–0.050 µg/mL for each target pesticide. Practical LOQs were chosen based on preliminary instrumental sensitivity, and, subsequently, accuracy was evaluated spiking on tomato sauce blank samples at the selected LOQ level (ATs; TeA: 75 µg/kg; ALT, AME, AOH and TEN: 1.5 µg/kg; pesticides: 0.0025, 0.0050 and 0.010 mg/kg). A second validation level was performed at higher concentrations for both ATs (TeA: 375 µg/kg; ALT, AME, AOH and TEN: 7.5 µg/kg) and pesticides (0.010 mg/kg). For pesticide analysis, the multiresidue quantification LC-MS method described in the European Standard UNI EN 15662:2018 was applied [34]. The method was appropriately modified within the terms of the normative, and, for each analytic session, linearity was verified by preparing calibration curves across five concentration levels (0.0025, 0.0050, 0.0100, 0.0250 and 0.0500 mg/kg) for every target analyte. Additionally, two different positive controls, at concentration levels of 0.0025 and 0.0500 mg/kg were used to confirm the goodness of the recovery for each analytical session. The requirements for molecule identification were set as indicated in document SANTE/11312/2021 v2:

- a minimum of two ions with mass accuracy ≤ 5 ppm;
- a minimum S/N ≥ 3;
- a full overlapping of the analyte precursor peak and product ion peak;
- the same ion ratio of the standards (not necessary but additional support to identification).

The defined LOQ for each analyte was set as the lowest spike level meeting the identification and method performance criteria for recovery and precision. The LOD, calculated for each analyte according to ICH guidelines as one-third of the LOQ, was then confirmed by verifying that the signal-to-noise (S/N) ratio of the mathematically defined detection level was consistently ≥3 [35]. The precision of the method for each target pesticide was confirmed through the determination of the CV% for the spiked samples at low concentration (LOQ) and high concentration (0.0500 mg/kg). LODs, LOQs, precision and trueness (in terms of average recoveries) are shown in Tables S4 and S5 for pesticides and ATs, respectively. Method biases were corrected for pesticides following SANTE/11312/2021 v2 appendix E option 1, preparing calibrations standards in an extract of a blank sample of the same matrix. For ATs, option 4 of appendix E was chosen, the use of ILISs. Quantitation was reported in µg/kg for ATs and in mg/kg for pesticides in accordance with the measurement units reported for indicative levels in Recommendation (EU) 553/2022 and MRLs in Regulation (EC) No 396/2005, respectively.

### 5.6. Dietary Exposure Assessment to Alternaria Toxins from Tomato Products

#### 5.6.1. Approach

For exposure assessment, analytical results below the LOD were imputed as LOD, using the substitution approach for treating left-censored data, commonly known as the “upper bound” (UB) [60]. The percentage of left-censored data was also indicated for the evaluations (Table S6). Due to the unavailability of properly defined toxicological reference values (only the TTC are defined for ATs), a traditional exposure assessment (point estimate), also known as “deterministic approach”, was used for this assessment, considering worst case estimate/scenario for the general population and subgroups.

#### 5.6.2. Hypothesis and Exposure Duration

Data on food consumption of tomato-based products (g/day) were obtained from the consultation of three tools. Using the FAOSTAT collaborative platform, consumption data for the general world population, the European population and the Italian population were obtained from 2010 to 2021, and the mean value was calculated [61]. The study on food consumption in Italy (SCAI) from 2005 to 2006 that was conducted by the National Research Institute for Food and Nutrition (INRAN) made it possible to obtain consumption data in the total population (both medium consumer and high consumer, understood as 95th percentile, P-95 and 99th percentile, P-99) [62]. Dietary exposure was estimated for the female and male populations. As in most exposure assessment studies, the present discussion takes into consideration the eating habits of consumers only, i.e., “subjects who consumed at least one item within the food category on at least one eating occasion during the survey”. Furthermore, for this assessment, chronic (long-life) exposure was considered, intended as “a long-term constant or intermittent exposure to a substance which may have an impact on health over time” (EFSA Glossary). Finally, the FoodEx database developed by EFSA was used for consumption in subgroups of the Italian population (infants, other children, toddlers, adolescents and older adults) [63]. For the exposure calculation, the data obtained were converted into g/kg bw day, using an average human weight of 60.0 kg, and, for population subclasses, the reference weights indicated in FoodEx. Where homologous data from population groups are present (e.g., the Italian population), they are comparable (SD < 1.0) and complementary (Table S7).

## Figures and Tables

**Table 1:** Validation parameters for *Alternaria* toxins and pesticides: summary.

Target Analyte		LOD	LOQ	Recovery Range Level 1 (%) *	Recovery Range Level 2 (%) **	RSD_r_ Level 1 *	RSD_r_ Level 2 **
***Alternaria* toxins** (μg/kg)	TeA	23	75	110	91	6.0	6.5
	ALT	0.5	1.5	109	108	8.9	9.9
	AOH	0.5	1.5	112	96	4.3	7.3
	TEN	0.5	1.5	109	89	5.2	6.4
	AME	0.5	1.5	99	102	4.7	8.5
Pesticides *** (mg/kg)	Group 1	0.0008	0.0025	60–133	74–112	3.2–25	2.2–25
	Group 2	0.0015	0.0050	74–119	71–113	-	4.5–27
	Group 3	0.0030	0.0100	-	92–111	-	12–14

* *Alternaria* toxins: LOQ; Pesticides: LOQ; ** *Alternaria* toxins: LOQ x5; Pesticides: 0.010 mg/kg; *** Analytes with one validation parameter outside the ranges reported in SANTE\11312\2021 v2 were considered for qualitative determination only.

**Table 2:** Occurrence of most common *Alternaria* toxins in tomato sauces (μg/kg).

TeA		AME		AOH		Refs.
Range	Average *(Median)*	Range	Average *(Median)*	Range	Average *(Median)*	
4–144	40 *(35)*	1–4	NR	4–10	NR	Noser et al., 2011 ^a^ [38]
52–460	200	1.2–7.4	2.5	6.1–25	13	Hickert et al., 2015 [39]
66–462	202 *(143)*	ND-7.8	3.8 *(3.0)*	ND-25	16 *(17)*	Lopez et al., 2015 [40]
7.7–330.6	84.3 *(64.1)*	ND-3.8	0.6 *(0.5)*	ND-41.6	2.7 *(0.8)*	Walravens et al., 2016 [41]
ND-198	18.6 *(<LOD)*	ND	ND	ND	ND	Bertuzzi et al., 2021 [37]
5.8–485	86.1	ND	ND	ND	ND	Lattanzio et al., 2021 [32]
75–379 ^b^	143	3.0–5.6	4.3	1.5–18	4.8	This work
75–517 ^c^	160	2.2–7.1	3.9	1.6–27	7.4	

^a^: only considering tomato sauces; ^b^: organic products; ^c^: conventional products.

## Data Availability

The original contributions presented in this study are included in this article and the Supplementary Materials. Further inquiries can be directed to the corresponding authors.

## Supplementary Materials

The following supporting information can be downloaded at: https://www.mdpi.com/article/10.3390/toxins17010012/s1, Table S1. Pesticides analytical standards; Table S2. LC-MS parameters for *Alternaria* toxins; Table S3. LC-HRMS parameters for pesticides; Table S4. LOD, LOQ, average recovery, and precision at low and high concentrations for pesticides; Table S5. LOD, LOQ, average recovery, and precision at low and high concentrations for *Alternaria* toxins; Table S6. Exposure assessment and risk characterization of *Alternaria* toxins in tomato sauce samples; Table S7. Consumption data tomato sauces/puree.

## References

[1] Gómez-Romero M., Arráez-Román D., Segura-Carretero A., Fernández-Gutiérrez A. (2007). Analytical Determination of Antioxidants in Tomato: Typical Components of the Mediterranean Diet. J. Sep. Sci..

[2] Tiwari J.K., Tusar B., Nagendra R., Suresh R.Y., Manish K.S., Singh P.M. (2021). Tomato Breeding for Processing in India: Current Status and Prospects. Veg. Sci..

[3] Van de Perre E., Deschuyffeleer N., Jacxsens L., Vekeman F., Van Der Hauwaert W., Asam S., Rychlik M., Devlieghere F., De Meulenaer B. (2014). Screening of Moulds and Mycotoxins in Tomatoes, Bell Peppers, Onions, Soft Red Fruits and Derived Tomato Products. Food Control.

[4] Meena M., Samal S. (2019). Alternaria Host-Specific (HSTs) Toxins: An Overview of Chemical Characterization, Target Sites, Regulation and Their Toxic Effects. Toxicol. Rep..

[5] European Food Safety Agency (EFSA) (2011). Scientific Opinion on the Risks for Animal and Public Health Related to the Presence of Alternaria Toxins in Feed and Food. EFSA J..

[6] Arcella D., Eskola M., Gómez Ruiz J.A. (2016). Dietary Exposure Assessment to Alternaria Toxins in the European Population. EFSA J..

[7] European Commission Commission Recommendation (EU) 2022/553 of 5 April 2022 on Monitoring the Presence of Alternaria Toxins in Food. https://eur-lex.europa.eu/legal-content/EN/TXT/PDF/?uri=CELEX:32022H0553.

[8] Rizwana H., Bokahri N.A., Alsahli S.A., Al Showiman A.S., Alzahrani R.M., Aldehaish H.A. (2021). Postharvest Disease Management of Alternaria Spots on Tomato Fruit by Annona Muricata Fruit Extracts. Saudi J. Biol. Sci..

[9] Martinko K., Ivanković S., Lazarević B., Đermić E., Đermić D. (2022). Control of Early Blight Fungus (*Alternaria alternata*) in Tomato by Boric and Phenylboronic Acid. Antibiotics.

[10] Slathia S., Sharma Y.P., Hakla H.R., Urfan M., Yadav N.S., Pal S. (2021). Post-Harvest Management of Alternaria Induced Rot in Tomato Fruits with Essential Oil of *Zanthoxylum Armatum* DC. Front. Sustain. Food Syst..

[11] Nehela Y., Mazrou Y.S.A., Taha N.A., Elzaawely A.A., Xuan T.D., Makhlouf A.H., El-Nagar A. (2023). Hydroxylated Cinnamates Enhance Tomato Resilience to *Alternaria alternata*, the Causal Agent of Early Blight Disease, and Stimulate Growth and Yield Traits. Plants.

[12] Schmey T., Tominello-Ramirez C.S., Brune C., Stam R. (2024). Alternaria Diseases on Potato and Tomato. Mol. Plant Pathol..

[13] European Commission Regulation (EC) No 396/2005 of the European Parliament and of the Council of 23 February 2005 on Maximum Residue Levels of Pesticides in or on Food and Feed of Plant and Animal Origin and Amending Council Directive 91/414/EEC. https://eur-lex.europa.eu/legal-content/EN/TXT/PDF/?uri=CELEX:32005R0396.

[14] European Commission Commission Implementing Regulation (EU) 2021/1165 of 15 July 2021 Authorising Certain Products and Substances for Use in Organic Production and Establishing Their Lists. https://eur-lex.europa.eu/legal-content/EN/TXT/?uri=CELEX%3A02021R1165-20231115.

[15] European Commission Commission Directive of 14 May 1991 on Infant Formulae and Follow-On Formulae. https://eur-lex.europa.eu/legal-content/EN/TXT/PDF/?uri=CELEX:31991L0321.

[16] European Commission Regulation (EU) No 609/2013 of the European Parliament and of the Council of 12 June 2013 on Food Intended for Infants and Young Children, Food for Special Medical Purposes, and Total Diet Replacement for Weight Control and Repealing Council Directive 92/52/EEC, Commission Directives 96/8/EC, 1999/21/EC, 2006/125/EC and 2006/141/EC, Directive 2009/39/EC of the European Parliament and of the Council and Commission Regulations (EC) No 41/2009 and (EC) No 953/2009. https://eur-lex.europa.eu/legal-content/EN/TXT/PDF/?uri=CELEX:02013R0609-20230321.

[17] Smaoui S., Agriopoulou S., D’Amore T., Tavares L., Mousavi Khaneghah A. (2022). The Control of *Fusarium* Growth and Decontamination of Produced Mycotoxins by Lactic Acid Bacteria. Crit. Rev. Food Sci. Nutr..

[18] Wu W., Huang X., Liang R., Guo T., Xiao Q., Xia B., Wan Y., Zhou Y. (2023). Determination of 63 Mycotoxins in Grain Products by Ultrahigh-Performance Liquid Chromatography Coupled with Quadrupole-Orbitrap Mass Spectrometry. Food Control.

[19] Pallarés N., Font G., Mañes J., Ferrer E. (2017). Multimycotoxin LC–MS/MS Analysis in Tea Beverages after Dispersive Liquid–Liquid Microextraction (DLLME). J. Agric. Food. Chem..

[20] Wong J.W., Wang J., Chow W., Carlson R., Jia Z., Zhang K., Hayward D.G., Chang J.S. (2018). Perspectives on Liquid Chromatography-High-Resolution Mass Spectrometry for Pesticide Screening in Foods. J. Agric. Food. Chem..

[21] Wang J., Leung D. (2009). Applications of Ultra-Performance Liquid Chromatography Electrospray Ionization Quadrupole Time-of-Flight Mass Spectrometry on Analysis of 138 Pesticides in Fruit- and Vegetable-Based Infant Foods. J. Agric. Food. Chem..

[22] Kmellár B., Abrankó L., Fodor P., Lehotay S.J. (2010). Routine Approach to Qualitatively Screening 300 Pesticides and Quantification of Those Frequently Detected in Fruit and Vegetables Using Liquid Chromatography Tandem Mass Spectrometry (LC-MS/MS). Food Addit. Contam. Part A.

[23] Li R., Li M., Wang T., Wang T., Chen J., Francis F., Fan B., Kong Z., Dai X. (2020). Screening of Pesticide Residues in Traditional Chinese Medicines Using Modified QuEChERS Sample Preparation Procedure and LC-MS/MS Analysis. J. Chromatogr. B.

[24] Tienstra M., Portolés T., Hernández F., Mol J.G.J. (2015). Fast Gas Chromatographic Residue Analysis in Animal Feed Using Split Injection and Atmospheric Pressure Chemical Ionisation Tandem Mass Spectrometry. J. Chromatogr. A.

[25] Pico Y., Alfarhan A.H., Barcelo D. (2020). How Recent Innovations in Gas Chromatography-Mass Spectrometry Have Improved Pesticide Residue Determination: An Alternative Technique to Be in Your Radar. TrAC Trends Anal. Chem..

[26] Keshet U., Goldshlag P., Amirav A. (2017). Pesticide Analysis by Pulsed Flow Modulation GCxGC-MS with Cold EI—An Alternative to GC-MS-MS. Anal. Bioanal. Chem..

[27] Eker F.Y., Muratoglu K., Ozturk M., Cetin B., Buyukunal S.K. (2023). Determination of Multimycotoxin in Cereal-Based Products Sold in Open-Air Markets. Foods.

[28] Tang Y.Y., Lin H.Y., Chen Y.C., Su W.T., Wang S.C., Chiueh L.C., Shin Y.C. (2012). Development of a Quantitative Multi-Mycotoxin Method in Rice, Maize, Wheat and Peanut Using UPLC-MS/MS. Food Anal. Methods.

[29] Solfrizzo M., Gambacorta L., Bibi R., Ciriaci M., Paoloni A., Pecorelli I. (2018). Multimycotoxin Analysis by LC-MS/MS in Cereal Food and Feed: Comparison of Different Approaches for Extraction, Purification, and Calibration. J. AOAC Int..

[30] Rossi F., Gallo A., Bertuzzi T. (2020). Emerging Mycotoxins in the Food Chain. Mediterr. J. Nutr. Metab..

[31] Patriarca A. (2016). Alternaria in Food Products. Curr. Opin. Food Sci..

[32] Lattanzio V.M.T., Verdini E., Sdogati S., Bibi R., Ciasca B., Pecorelli I. (2021). Monitoring *Alternaria* Toxins in Italian Food to Support Upcoming Regulation. Food Addit. Contam Part B Surveill.

[33] European Commission Directorate-General for Health and Food Safety (DG SANTE). Guidance Document SANTE 11312/2021 v2—Analytical Quality Control and Method Validation Procedures for Pesticide Residues Analysis in Food and Feed. Version 2 implemented by 01/01/2024. https://food.ec.europa.eu/document/download/d4786faf-c574-4222-a5c6-45086b3920b8_en?filename=pesticides_mrl_guidelines_wrkdoc_2021-11312.pdf.

[34] (2018). Foods of Plant Origin—Multimethod for the Determination of Pesticide Residues Using GC- and LC-Based Analysis Following Acetonitrile Extraction/Partitioning and Clean-up by Dispersive SPE—Modular QuEChERS-Method.

[35] ICH Expert Working Group (2022). ICH Guideline Q2(R2) on Validation of Analytical Procedures.

[36] European Commission Commission Implementing Regulation (EU) 2023/2782 of 14 December 2023 Laying down the Methods of Sampling and Analysis for the Control of the Levels of Mycotoxins in Food and Repealing Regulation (EC) No 401/2006 (Text with EEA Relevance) Text with EEA Relevance. http://data.europa.eu/eli/reg_impl/2023/2782/2024-03-24.

[37] Bertuzzi T., Rastelli S.E., Pietri A., Giorni P. (2021). *Alternaria* Toxins in Tomato Products in Northern Italy in the Period 2017–2019. Food Addit. Contam. Part B.

[38] Noser J., Schneider P., Rother M., Schmutz H. (2011). Determination of Six Alternaria Toxins with UPLC-MS/MS and Their Occurrence in Tomatoes and Tomato Products from the Swiss Market. Mycotoxin Res..

[39] Hickert S., Bergmann M., Ersen S., Cramer B., Humpf H.-U. (2015). Survey of Alternaria Toxin Contamination in Food from the German Market, Using a Rapid HPLC-MS/MS Approach. Mycotoxin Res..

[40] López P., Venema D., de Rijk T., de Kok A., Scholten J.M., Mol H.G.J., de Nijs M. (2016). Occurrence of Alternaria Toxins in Food Products in the Netherlands. Food Control.

[41] Walravens J., Mikula H., Rychlik M., Asam S., Devos T., Njumbe Ediage E., Mavungu J.D.D., Jacxsens L., Van Landschoot A., Vanhaecke L. (2016). Validated UPLC-MS/MS Methods to Quantitate Free and Conjugated *Alternaria* Toxins in Commercially Available Tomato Products and Fruit and Vegetable Juices in Belgium. J. Agric. Food. Chem..

[42] Abd-Elhaleem Z.A. (2020). Pesticide Residues in Tomato and Tomato Products Marketed in Majmaah Province, KSA, and Their Impact on Human Health. Environ. Sci. Pollut. Res..

[43] Balkan T., Kara K. (2023). Pesticide Residues in Sauce Manufactured from Agricultural Products. Int. J. Agric. Environ. Food Sci..

[44] European Commission Directive 2000/60/EC of the European Parliament and of the Council of 23 October 2000 Establishing a Framework for Community Action in the Field of Water Policy. https://eur-lex.europa.eu/legal-content/EN/TXT/?uri=CELEX%3A02000L0060-20141120.

[45] European Environment Agency (EEA) Pesticides in Rivers, Lakes and Groundwater in Europe. https://www.eea.europa.eu/en/analysis/indicators/pesticides-in-rivers-lakes-and?activeAccordion=546a7c35-9188-4d23-94ee-005d97c26f2b..

[46] European Commission Regulation Dimethomorph. https://ec.europa.eu/food/plant/pesticides/eu-pesticides-database/backend/api/active_substance/download/1450.

[47] Smaoui S., D’Amore T., Tarapoulouzi M., Agriopoulou S., Varzakas T. (2023). Aflatoxins Contamination in Feed Commodities: From Occurrence and Toxicity to Recent Advances in Analytical Methods and Detoxification. Microorganisms.

[48] Serafimova R., Coja T., Kass G.E.N. (2021). Application of the Threshold of Toxicological Concern (TTC) in Food Safety: Challenges and Opportunities. Front. Toxicol..

[49] EFSA (2019). Guidance on the Use of the Threshold of Toxicological Concern Approach in Food Safety Assessment. EFSA J..

[50] Pavicich M.A., De Boevre M., Vidal A., Mikula H., Warth B., Marko D., De Saeger S., Patriarca A. (2023). Natural Occurrence, Exposure Assessment & Risk Characterization of Alternaria Mycotoxins in Apple By-Products in Argentina. Expo. Health.

[51] Pompa C., D’Amore T., Miedico O., Preite C., Chiaravalle A.E. (2021). Evaluation and Dietary Exposure Assessment of Selected Toxic Trace Elements in Durum Wheat (*Triticum durum*) Imported into the Italian Market: Six Years of Official Controls. Foods.

[52] Fernández-Blanco C., Font G., Ruiz M.-J. (2016). Role of Quercetin on Caco-2 Cells against Cytotoxic Effects of Alternariol and Alternariol Monomethyl Ether. Food Chem. Toxicol..

[53] Crudo F., Varga E., Aichinger G., Galaverna G., Marko D., Dall’Asta C., Dellafiora L. (2019). Co-Occurrence and Combinatory Effects of Alternaria Mycotoxins and Other Xenobiotics of Food Origin: Current Scenario and Future Perspectives. Toxins.

[54] Rizzati V., Briand O., Guillou H., Gamet-Payrastre L. (2016). Effects of Pesticide Mixtures in Human and Animal Models: An Update of the Recent Literature. Chem. Biol. Interact..

[55] Eze U.A., Huntriss J., Routledge M.N., Gong Y.Y. (2018). Toxicological Effects of Regulated Mycotoxins and Persistent Organochloride Pesticides: In Vitro Cytotoxic Assessment of Single and Defined Mixtures on MA-10 Murine Leydig Cell Line. Toxicol. In Vitro.

[56] Eze U.A., Huntriss J., Routledge M.N., Gong Y.Y., Connolly L. (2019). The Effect of Individual and Mixtures of Mycotoxins and Persistent Organochloride Pesticides on Oestrogen Receptor Transcriptional Activation Using in Vitro Reporter Gene Assays. Food Chem. Toxicol..

[57] Tadee A., Mahakunakorn P., Porasuphatana S. (2020). Oxidative Stress and Genotoxicity of Co-Exposure to Chlorpyrifos and Aflatoxin B1 in HepG2 Cells. Toxicol. Ind. Health.

[58] Cedergreen N. (2014). Quantifying Synergy: A Systematic Review of Mixture Toxicity Studies within Environmental Toxicology. PLoS ONE.

[59] Fu Y., Yin S., Zhao C., Fan L., Hu H. (2022). Combined Toxicity of Food-Borne Mycotoxins and Heavy Metals or Pesticides. Toxicon.

[60] EFSA (2010). Management of Left-Censored Data in Dietary Exposure Assessment of Chemical Substances. EFSA J..

[61] FAO—Food and Agriculture Organization of the United Nations Statistics Division FAOSTAT—Food and Agricultural Databases. https://www.fao.org/faostat/en/#data/FBS.

[62] Leclercq C., Arcella D., Piccinelli R., Sette S., Le Donne C. (2009). The Italian National Food Consumption Survey INRAN-SCAI 2005–06: Main Results in Terms of Food Consumption. Public Health Nutr..

[63] EFSA. https://www.efsa.europa.eu/en/supporting/pub/en-7900.

