# Supplementary material for: Assessment of Alternaria Toxins and Pesticides in Organic and Conventional Tomato Products: Insights into Contamination Patterns and Food Safety Implications
Source: Toxins (Basel). 2024 Dec 29;17(1):12. doi: 10.3390/toxins17010012 (PMC11769159; doi:10.3390/toxins17010012)
Supplement: Supplementary file 1 [file toxins-17-00012-s001.zip › toxins-3373703-supplementary.pdf]

Table S1. Pesticides Analytical Standards

Analyte	CAS	Analyte	CAS	Analyte	CAS	Analyte	CAS	Analyte	CAS
3-Hydroxy carbofuran[1,6]	6655-82-6	Deltamethrin[1]	5298-63-5	Fenthion Sulfone[1,5]	376-42-0	Methacrifos[1]	6260-77-9	Prothiofos[1]	34643-46-4
Acephate[1]	30560-19-1	Demeton-S-Methyl-sulfone[1,5]	17040-19-6	Fenthion Sulfoxide[1,5]	376-4-9	Methamidophos[1,5]	10265-92-6	Pymetrozine[1]	2332-89-0
Acetamiprid[1]	135410-20-7	Diazinon[1,5]	333-41-5	Fenvalerate[1,8]	5630-58-1	Methidathion[1,5]	950-37-8	Pyraclostrobin[2]	175013-18-0
Aclonifen[3]	74070-46-5	Dichlorvos[1,5]	62-73-7	Fipronil[1]	20068-37-3	Methiocarb[1,5]	2032-65-7	Pyrazophos[1,2]	13457-18-6
Aldicarb[1,5,6]	116-06-03	Diethofencarb[2]	87130-20-9	Flonicamid[1]	58062-67-0	Methiocarb Solfone[1,5]	2179-25-1	Pyridaben[1,5]	96489-7-3
Aldicarb Sulfone[1]	1646-88-4	Difenoconazole[2]	119446-68-3	Flubendiamide[1]	27245-65-7	Methiocarb Sulfoxide[1,5]	2635-0-0	Pyridalyl[1]	790-8-6
Aldicarb Sulfoxide[1]	1646-87-3	Diflubenzuron[1]	35367-38-5	Flucythrinate[1]	7024-77-5	Methomyl[1,5]	6752-77-5	Pyrimethanil[2]	532-28-0
Ametoctradin[2]	865318-97-4	Dimethoate[1]	60-51-5	Fludioxonil[2]	131341-86-1	Methoxyfenozide[1]	6050-58-4	Pyriproxyfen[1]	95737-68-
Avermectin 8,9-Z[1,5]	3665-89-7	Dimethomorph[2]	110488-70-5	Flufenoxuron[1,5]	0463-69-8	Metrafenone[2]	220899-03-6	Quinoxyfen[2]	24495-8-7
Avermectin B1a[1,5]	6595-55-3	Diniconazole[2]	83657-24-3	Fluopicolide[2]	239110-15-7	Monocrotophos[1,5]	6923-22-4	Spinetoram J[1]	8766-40-
Avermectin B1b[1,5]	6595-56-4	Disulfoton[1,5]	0298-04-04	Fluopyram[2]	658066-35-4	Myclobutanil[2]	8867-89-0	Spinetoram L[1]	8766-5-0
Azinphos Methyl[1]	86-50-0	Emamectin benzoate B1a[1]	55569-9-8	Fluquinconazole[2]	136426-54-5	Omethoate[1,5]	1113-02-06	Spinosyn A[1]	3929-60-7
Azoxystrobin[2]	131860-33-8	EPN[1,5]	204-64-5	Flusilazole[2]	85509-19-9	Oxadixyl[2]	0296-09-03	Spinosyn D[1]	3929-63-0
Benfuracarb[1]	82560-54-1	Epoxiconazole[2]	133855-98-8	Flutriafol[2]	76674-21-0	Oxamyl[1,5,6]	2335-22-0	Spirodiclofen[1]	48477-7-8
Bifenthrin[1,5]	82657-04-3	Ethion[1,5]	563-12-2	Fluvalinate Tau[1,5]	0285-06-9	Oxydemeton Methyl[1]	0030-02-02	Spiromesifen[1]	283594-90-1
Bitertanol[2]	55179-31-2	Ethirimol[2]	23947-60-6	Fluxapyroxad[2]	907204-3-3	Paclobutrazol[7]	76738-60-0	Spirotetramat[1]	20333-25-1
Boscalid[2]	188425-85-6	Etofenprox[1]	80844-07-1	Formetanate (Hydrochloride)[1,5]	23422-53-9	Paraoxon Methyl[1]	950-35-6	Spirotetramat-enol[1]	20332-38-3
BTS 44595[2,4]	39520-94-8	Etoxazole[5]	153233-91-1	Fosthiazate[1,6]	98886-44-3	Parathion[1,5]	56-38-2	Spiroxamine[2]	118134-30-8
BTS 44596[2,4]	39542-32-8	Famoxadone[2]	3807-57-3	Furathiocarb[1]	65907-30-4	Parathion Methyl[1,5]	298-00-0	Sulfoxaflor[1]	946578-00-3

Bupirimate[2]	4483-43-6	Fenamidone[2]	161326-34-7	Haloxyfop[3]	69806-34-4	Penconazole[2]	66246-88-6	Tebuconazole[2]	107534-96-3
Buprofezin[1,5]	69327-76-0	Fenamiphos[6]	22224-92-6	Hexaconazole[2]	79983-71-4	Pencycuron[2]	66063-05-6	Tebufenozide[1]	240-23-8
Carbaryl[1,7]	63-25-2	Fenamiphos Sulfone[6]	3972-44-8	Hexythiazox[5]	78587-05-0	Pencycuron PB Amine[2]	66063-15-8	Tebufenpyrad[5]	968-77-3
Carbendazim[2]	0605-2-7	Fenamiphos Sulfoxide[6]	3972-43-7	Imazalil[2]	35554-44-0	Pendimethalin[3]	40487-42-	Teflubenzuron[1]	832-8-0
Carbofuran[1,6]	563-66-2	Fenarimol[2]	6068-88-9	Imidacloprid[1]	138261-43-3	Phenthoate[1,5]	2597-03-7	Terbuthylazine[3]	5915-41-3
Carbosulfan[1,6]	55285-4-8	Fenazaquin[5]	120928-09-8	Indoxacarb[1]	144171-61-9	Pholpet[2]	33-07-3	Tetraconazole[2]	112281-77-3
Chlorantraniliprole[1]	500008-45-7	Fenbuconazole[2]	114369-43-6	Iprodione[2,6]	36734-9-7	Phosalone[1,5]	2310-17-0	TFNA-AM[1]	58062-7-6
Chlorpyrifos[1]	2921-88-2	Fenbutatin Oxide[5]	13356-08-6	Iprovalicarb[2]	140923-17-7	Phosmet[1,5]	732-11-6	Thiabendazole[2]	48-79-8
Chlorpyrifos Methyl[1,5]	5598-3-0	Fenhexamid[2]	126833-17-8	Isoprothiolane[2,7]	5052-35-1	Pirimicarb[1]	23103-98-2	Thiacloprid[1]	988-49-9
Clofentezine[5]	745-24-5	Fenitrothion[1]	122-14-5	Kresoxim-Methyl[2]	43390-89-0	Pirimiphos Methyl[1,5]	29232-93-7	Thiamethoxam[1]	5379-23-4
Clothianidin[1]	20880-92-5	Fenoxycarb[1]	72490-01-8	Lambda-Cyalothrin[1]	91465-08-6	Prochloraz[2]	67747-09-5	Thiodicarb[1]	59669-26-0
Coumaphos[1,5]	56-72-4	Fenpropathrin[1,5]	39515-41-8	Linuron[3]	330-55-2	Procymidone[2]	32809-6-8	Tiophanate Methyl[2]	23564-05-08
Cyantraniliprole[1]	736994-63-1	Fenpropidin[2]	67306-00-7	Lufenuron[1,2,5]	03055-07-8	Profenofos[1,5]	41198-08-7	Tolclofos Metile[2]	57018-04-9
Cyazofamid[1]	206-88-3	Fenpropimorph[2]	67564-91-4	Malaoxon[4]	1634-78-2	Propamocarb[2]	24579-73-5	Triadimefon[2]	432-43-3
Cyflufenamid[2]	80409-60-3	Fenpyrazamine[2]	473798-59-3	Malathion[1,5]	121-75-5	Propargite[5]	232-35-8	Triadimenol[2]	55219-65-3
Cyfluthrin[1]	68359-37-5	Fenpyroximate[5]	34098-6-6	Mandipropamid[2]	374726-62-2	Propiconazole[2]	60207-90-1	Triazophos[1,5,6]	2407-47-8
Cymoxanil[2]	57996-95-7	Fenthion[1,5]	55-38-9	Mepanipyrim[2]	110235-47-7	Propyzamide[3]	23950-58-5	Tricyclazole[2]	484-78-2
Cypermethrin[1]	52315-07-8	Fenthion Oxon[1,5]	6552-13-2	Metaflumizone E[1]	852403-68-0	Proquinazid[2]	89278-2-4	Trifloxystrobin[2]	141517-21-7
Cyproconazole[2]	94361-06-05	Fenthion Oxon Sulfone[1,5]	4086-35-2	Metaflumizone Z[1]	39970-56-2	Prosulfocarb[3]	52888-80-9	Triflumuron[1]	64628-44-0
Cyprodinil[2]	121552-61-2	Fenthion Oxon Sulfoxide[1,5]	6552-13-2	Metalaxyl[2]	57837-19-1	Prothioconazole Desthio[2]	20983-64-4	Trifluralin[3]	1582-09-8

[1] Insecticide; [2] Fungicide; [3] Herbicide; [4] Metabolite; [5] Acaricide, [6] Nematicide, [7] Plant growth regulator, [8]Ixodicide.
Reference: Tomlin, Clive; The pesticide manual: a world compendium, 2021

Table S2. LC-MS parameters for *Alternaria* Toxins

Analyte	RT (min)	Precursor ion (m/z)	Product ions (m/z)	DP (V)	EP (V)	CE (V)	CXP (V)
TeA	2.71	196	139 (Q)	-72	-10	-27	-15
			112 (q)	-72	-10	-32	-13
TeA $^{13}C_2$	2.69	198	141	-72	-10	-27	-15
ALT	6.80	291	248 (Q)	-120	-10	-21	-20
			228 (q)	-120	-10	-37	-20
ALT d_6	6.80	297	203	-120	-10	-47	-18
AOH	7.57	257	215 (Q)	-120	-10	-35	-4
			147 (q)	-120	-10	-42	-13
AOH d_3	7.57	260	218	-120	-10	-35	-13
TEN	7.94	413	141 (Q)	-120	-10	-22	-13
			271 (q)	-120	-10	-27	-13
TEN d_3	7.94	416	274	-120	-10	-22	-13
AME	9.00	271	256 (Q)	-61	-10	-31	-13
			228 (q)	-61	-10	-40	-11
AME d_3	9.00	274	256	-61	-10	-31	-20

Table S3. LC-HRMS parameters for pesticides

Column	Luna Omega Polar C18 (100 mm x 2.1 mm particle size 1.6 µm) + pre column
Acquisition mode	SWATH
Ionization mode	ESI +
CUR (psi)	35
IS (V)	5300
TEM (°C)	450
GS1 (psi)	50
GS2 (psi)	55
Injection Volume (µL)	5
Oven temperature (°C)	40

Chromatographic Run

STEP	Time (min)	Flow (mL/min)	FM A (%)	FM B (%)
0	0	0.4	95	5
1	0.5	0.4	95	5
2	1	0.4	80	20
3	2.5	0.4	60	40
4	4.0	0.4	45	55
5	5.5	0.4	45	55
6	7.5	0.4	35	65
7	8.5	0.4	35	65
8	9.5	0.4	30	70
9	10.5	0.4	25	75
10	11	0.4	15	85
11	13.5	0.4	0	100
12	15.5	0.4	0	100
13	15.1	0.4	95	5
14	20	0.4	95	5

Precursors and fragment exact masses

**Target analyte**	**Precursor (m/z)**	**Fragment (m/z)**	**Target analyte**	**Precursor (m/z)**	**Fragment (m/z)**
3-Hydroxycarbofuran	238.107	107.050	Hexaconazole	314.082	70.0410
		163.075			158.976
Acephate	184.019	142.99	Hexythiazox	353.109	168.058
		124.982			228.024
Acetamiprid	223.075	126.009	Imazalil	297.056	158.977
		149.023			255.010
Aclonifen	265.037	248.033	Imidacloprid	256.06	209.058
		182.059			175.097
Aldicarb	208.111	89.042	Indoxacarb	528.078	150.011
		61.011			218.043
Aldicarb-sulfoxide	207.079	89.041	Iprodione	330.041	101.034
		69.057			99.018
Aldicarb Sulfone	240.101	86.061	Iprovalicarb	321.217	119.085
		81.001			116.071
Ametoctradin	276.218	176.094	Isoprothiolane	291.072	188.967
		177.101			231.014
Avermectin 8-9 Z	890.526	305.204	Kresoxim-Methyl	314.139	116.051
		567.321			131.073

Avermectin B1a	890.526	305.212	Lambda-Cyalothrin	467.139	225.029
		567.336			450.107
Avermectin B1b	876.510	291.194	Linuron	249.019	159.971
		553.319			132.96
Azinphos Methyl	318.013	132.044	Lufenuron	510.986	158.042
		124.982			141.014
Azoxystrobin	404.124	344.105	Malaoxon	315.066	99.007
		372.098			127.038
Bifenthrin	440.160	405.285	Malathion	331.043	99.006
		181.101			124.981
Bitertanol	338.186	99.080	Mandipropamid	412.131	125.017
		70.041			328.11
Boscalid	343.040	307.062	Mepanipyrim	224.118	106.066
		139.989			209.096
BTS 44595	325.027	284.019	Metaflumizone	507.125	178.049
		129.101			287.082
BTS 44596	353.022	70.028	Metalaxyl	280.154	160.112
		308.000			192.138
Bupirimate	317.164	166.099	Methacrifos	241.029	124.981
		108.013			209.002
Buprofezin	306.163	106.066	Methamidophos	142.009	94.004
		201.105			124.982
Carbaryl	202.086	145.065	Methidathion	302.969	85.039
		127.054			145.006
Carbendazim	192.077	160.05	Methiocarb	226.09	121.063
		132.055			122.072
Carbofuran	222.112	123.044	Methiocarb Sulfone	275.106	122.073
		165.091			201.058
Carbosulfan	381.221	118.067	Methiocarb Sulfoxide	242.085	122.073
		160.114			185.064
CGA 304075	242.129	93.057	Methomyl	163.054	73.000
		108.08			88.021
Chlorantraniliprole	481.978	283.923	Methoxyfenozide	369.217	149.059
		450.939			133.066
Chlorpyrifos	349.934	199.926	Metrafenone	409.065	209.081
		96.9530			226.97
Chlorpyrifos Methyl	321.902	124.982	Monocrotophos	224.068	127.015
		289.874			58.029
Clofentezine	303.02	138.008	Myclobutanil	289.121	70.041
		102.035			125.016
Clothianidin	250.016	131.966	Omethoate	214.03	77.038
		169.053			124.981
Coumaphos	363.022	226.992	Oxadixyl	279.134	149.023
		306.959			132.08
Cyantraniliprole	475.01	285.919	Oxamyl	237.102	72.044
		443.969			90.056
Cyazofamid	325.052	108.011	Oxydemeton Methyl	247.022	169.008
		266.038			109.005
Cyflufenamid	4131.28	241.039	Paclobutrazol	294.137	70.041
		295.086			125.016

Cyfluthrin	451.099	191.002	Paraoxon Methyl	248.032	202.039
		206.06			109.005
Cymoxanil	199.083	111.019	Parathion	292.040	235.977
		128.044			123.031
Cypermethrin	433.108	191.002	Parathion Methyl	264.009	124.981
		416.084			231.982
Cyproconazole	292.121	125.014	Penconazole	284.072	158.976
		70.040			70.041
Cyprodinil	226.134	93.059	Pencycuron	329.142	125.015
		108.081			218.073
Deltamehtrin	523.005	280.899	Pencycuron PB amine	210.104	125.01
		505.978			89.035
Demeton-S-Methyl-sulfone	263.017	169.008	Pendimethalin	282.145	212.065
		109.005			194.057
Diazinon	305.108	169.079	Phenthoate	321.038	135.043
		153.102			107.049
Dichlorvos	220.953	109.003	Pholpet	314.934	130.028
		78.994			201.962
Diethofencarb	268.154	124.038	Phosalone	367.994	181.000
		152.07			182.000
Difenoconazole	406.072	251.002	Phosmet	318.002	160.042
		337.04			133.029
Diflubenzuron	311.039	141.014	Pirimicarb	239.15	72.046
		158.04			182.127
Dimethoate	230.007	124.981	Pirimiphos Methyl	306.104	164.117
		170.97			108.055
Dimethomorph	388.131	301.063	Prochloraz	376.038	307.999
		165.055			308.000
Diniconazole	326.082	70.040	Procymidone	301.051	256.028
		158.977			284.024
Disulfoton	275.036	89.044	Profenofos	374.942	302.864
		61.012			304.862
Emamectin benzoate (B1a)	886.531	158.118	Propamocarb	189.160	102.054
		302.196			74.025
EPN	324.045	156.986	Propargite	368.189	175.111
		296.012			163.112
Epoxiconazole	330.080	121.044	Propiconazole	342.077	158.976
		123.025			69.070
Ethion	384.995	142.936	Propyzamide	256.029	172.954
		170.969			189.981
Ethirimol	210.160	140.109	Proquinazid	373.041	288.948
		98.062			271.921
Etofenprox	394.238	177.127	Prosulfocarb	252.142	91.054
		183.08			128.106
Etoxazole	360.177	141.015	Prothioconazole Desthio	312.066	125.014
		304.116			70.04
Famoxadone	392.160	238.107	Prothiofos	344.97	240.904
		195.082			160.955
Fenamidone	312.117	92.0510	Pymetrozine	218.104	105.044
		236.118			79.041
Fenamiphos	304.113	217.008	Pyraclostrobin	388.106	163.062

		201.984			164.07
Fenamiphos Sulfone	336.103	108.056	Pyrazophos	374.093	222.088
		188.047			194.056
Fenamiphos Sulfoxide	320.108	108.056	Pyridaben	365.145	147.116
		233.003			309.082
Fenarimol	331.040	268.052	Pyridalyl	489.975	108.96
		259.007			164.03
Fenazaquin	307.180	57.072	Pyrimethanil	200.118	183.092
		161.133			107.061
Fenbuconazole	337.121	125.014	Pyriproxyfen	322.144	96.044
		70.040			185.059
Fenbutatin Oxide	519.206	351.012	Quinoxyfen	308.004	196.98
		463.136			272.028
Fenhexamid	302.071	97.101	Spinetoram A	748.499	142.124
		55.055			98.098
Fenitrothion	278.025	124.982	Spinetoram B	760.499	142.122
		245.998			725.648
Fenoxycarb	302.139	88.039	Spinosyn A	732.468	142.122
		256.095			98.097
Fenpropathrin	350.175	125.096	Spinosyn D	746.484	142.122
		97.100			697.612
Fenpropidin	274.253	147.116	Spirodiclofen	411.112	313.039
		86.096			71.086
Fenpropimorph	304.263	147.118	Spiromesifen	371.222	273.148
		130.124			255.139
Fenpyrazamine	332.143	216.115	Spirotetramat	374.196	216.104
		189.091			270.152
Fenpyroximate	422.207	366.144	Spirotetramat-enol	302.175	216.102
		214.099			270.149
Fenthion	279.027	169.012	Spiroxamine	298.274	144.138
		105.069			100.113
Fenthion Oxon Sulfoxide	279.045	264.024	Sulfoxaflor	278.057	154.044
		104.062			174.051
Fenthion Oxon Sulfone	295.040	217.064	tau-Fluvalinate	503.134	181.064
		104.063			208.075
Fenthion Sulfone	311.017	124.981	Tebuconazole	308.152	70.04
		78.994			125.015
Fenthion Sulfoxide	295.022	109.004	Tebufenozide	353.222	133.064
		78.994			105.071
Fenthion Oxon	263.050	216.002	Tebufenpyrad	344.168	147.118
		231.025			145.054
Fenvalerate	437.163	167.062	Teflubenzuron	380.982	141.015
		181.064			158.041
Fipronil	453.973	367.950	Terbuthylazine	230.117	174.054
		436.945			132.032
Flonicamid	230.054	148.036	Tetraconazole	372.029	158.975
		203.042			70.04
Flubendiamide	683.031	273.934	TFNA-AM	191.043	148.036
		407.977			98.04
Flucythrinate	469.193	412.154	Thiabendazole	202.043	175.032
		199.093			131.06

Fludioxonil	266.074	229.041	Thiacloprid	253.031	126.01
		158.04			90.035
Flufenoxuron	489.044	158.041	Thiamethoxam	292.027	131.966
		141.014			181.056
Fluopicolide	382.973	172.954	Thiodicarb	355.056	88.021
		144.958			107.993
Fluopyram	397.054	173.019	Thiophanate Methyl	343.053	151.032
		208.012			160.05
Fluquinconazole	376.016	306.983	Tolclofos Methyl	300.962	174.971
		349.006			124.981
Flusilazole	316.108	165.071	Triadimefon	294.100	197.072
		247.074			69.070
Flutriafol	302.110	70.0420	Triadimenol	296.116	70.040
		123.025			99.080
Fluxapyroxad	382.097	342.085	Triazophos	314.072	162.066
		314.090			119.061
Formetanate	222.124	165.103	Tricyclazole	190.043	136.022
		120.045			163.032
Fosthiazate	284.054	104.016	Trifloxystrobin	409.137	186.052
		227.991			131.072
Furathiocarb	383.164	195.047	Triflumuron	359.04	156.021
		167.053			138.994
Haloxyfop	362.04	316.035			
		272.001			

SWATH method

Experiment N°	Scan type	Product of	Accumulati on time (sec)	TOF MS range (da)	DP	CE
1	TOF MS	-	0.05	100-950	50	10
2	Product ion	120-70	0.025	44-612	40	35
3	Product ion	169-200	0.025	44-225	50	35
4	Product ion	199-215	0.025	44-225	70	35
5	Product ion	214-225	0.025	50-235	60	35
6	Product ion	224-240	0.025	44-250	60	35
7	Product ion	239-250	0.025	50-250	70	35
8	Product ion	249-260	0.025	50-270	60	35
9	Product ion	259-270	0.025	50-280	45	35
10	Product ion	269-280	0.025	50-290	60	35
11	Product ion	279-290	0.025	50-300	60	35
12	Product ion	289-300	0.025	44-310	60	35
13	Product ion	299-305	0.020	50-315	80	35
14	Product ion	304-310	0.035	30-320	70	35
15	Product ion	309-315	0.025	50-325	70	35
16	Product ion	314-320	0.025	50-330	70	35
17	Product ion	319-330	0.025	50-330	60	35
18	Product ion	329-340	0.025	50-340	70	35
19	Product ion	339-350	0.025	40-350	70	35
20	Product ion	349-360	0.025	50-370	50	35
21	Product ion	359-370	0.025	50-380	70	35
22	Product ion	369-380	0.025	50-390	70	35
23	Product ion	379-400	0.025	50-410	70	35

24	Product ion	399-410	0.025	50-420	80	35
25	Product ion	409-430	0.025	50-440	80	35
26	Product ion	429-480	0.025	50-490	80	35
27	Product ion	479-530	0.025	50-540	80	35
28	Product ion	529-730	0.025	50-740	80	35
29	Product ion	729-950	0.025	50-950	90	35

Table S4. LOD, LOQ, average recovery, and precision at low and high concentrations for pesticides

Target Analyte	LOD (μg/kg)	LOQ (μg/kg)	Average Rec% LOQ (μg/kg)	Average Rec% (10 μg/kg)	RSD$_r$ LOQ (μg/kg)	RSD$_r$ (10 μg/kg)
3-hydroxycarbofuran	0.8	2.5	86	92	12	20
Acephate*	0.8	2.5	85	80	20	24
Acetamiprid	0.8	2.5	112	90	8.3	20
Aclonifen	1.5	5.0	87	99	8.1	8.1
Aldicarb	0.8	2.5	83	96	19	8.5
Aldicarb-sulfoxide	1.5	5.0	85	82	15	15
Aldoxycarb	0.8	2.5	106	93	10	6.8
Ametoctradin	1.5	5.0	119	92	15	11
Avermectin B1a	3.0	10	111	111	14	14
Avermectina 8-9 Z*	0.8	2.5	75	110	24	19
Avermectina B1b	3.0	10	92	92	12	12
Azinphos-methyl	0.8	2.5	97	97	9.4	12
Azoxystrobin	0.8	2.5	107	94	8.4	9.7
Bifenthrin	1.5	5.0	119	71	17	20
Bitertanol*	0.8	2.5	133	112	21	18
Boscalid	0.8	2.5	102	99	8.5	3.6
BTS 44595	0.8	2.5	96	77	20	13
BTS 44596	0.8	2.5	94	100	13	3.4
Bupirimate	0.8	2.5	105	95	10	9.8
Buprofezin	1.5	5.0	103	97	19	12
Carbaryl	0.8	2.5	100	93	11	13
Carbendazim*	1.5	5.0	91	84	18	23
Carbofuran*	0.8	2.5	124	94	11	18
Carbosulfan	0.8	2.5	94	91	14	8.3
Chlorantraniliprole	1.5	5.0	102	94	11	10
Chlorpyrifos	1.5	5.0	103	96	15	19
Chlorpyrifos-methyl	1.5	5.0	97	98	11	6.6
Clofentezine	1.5	5.0	101	95	12	11
Clothianidin	0.8	2.5	96	99	6.8	11
Coumaphos	0.8	2.5	99	101	13	10
Cyantraniliprole	0.8	2.5	103	94	11	2.2
Cyazofamid	0.8	2.5	102	97	11	6.3
Cyflufenamid	0.8	2.5	99	95	16	6.7
Cyfluthrin	1.5	5.0	116	79	20	11
Cymoxanil	1.5	5.0	74	83	20	19

Cypermethrin	1.5	5.0	110	95	20	20
Cyproconazole	0.8	2.5	100	97	3.3	1.8
Cyprodinil	0.8	2.5	105	90	9.5	14
Deltamethrin	1.5	5.0	89	97	15	4.5
Demeton-S-methyl-sulfone	0.8	2.5	106	91	12	13
Diazinon	0.8	2.5	105	99	16	7.4
Dichlorvos	0.8	2.5	108	94	9.2	12
Diethofencarb	0.8	2.5	103	98	6.9	6.6
Difenoconazole	0.8	2.5	104	97	14	8.5
Diflubenzuron	0.8	2.5	102	96	14	7.9
Dimethoate*	1.5	5.0	101	88	20	25
Dimethomorph	0.8	2.5	76	102	6.8	6.1
Diniconazole	0.8	2.5	92	99	8.1	7.3
Disulfoton	1.5	5.0	99	97	20	13
Emamectin benzoate	1.5	5.0	105	93	20	15
EPN	1.5	5.0	99	97	20	13
Epoxiconazole	0.8	2.5	105	98	11	5.0
Ethion	1.5	5.0	107	95	20	20
Ethirimol	1.5	5.0	100	80	9.4	20
Etofenprox	1.5	5.0	92	89	20	18
Etoxazole	1.5	5.0	103	96	15	4.6
Famoxadone*	0.8	2.5	104	93	25	12
Fenamidone*	0.8	2.5	121	102	14	7.8
Fenamiphos	0.8	2.5	101	98	16	10
Fenamiphos-sulfone	0.8	2.5	88	100	12	5.5
Fenamiphos-sulfoxide	0.8	2.5	117	98	13	8.1
Fenarimol	0.8	2.5	108	91	10	4.7
Fenazaquin	0.8	2.5	97	94	14	7.1
Fenbuconazole	0.8	2.5	86	95	20	8.4
Fenbutatin Oxide*	0.8	2.5	60	60	17	25
Fenhexamid	0.8	2.5	66	87	11	5.6
Fenitrothion*	0.8	2.5	67	111	23	18
Fenoxycarb	0.8	2.5	78	101	10	6.3
Fenpropathrin	0.8	2.5	110	109	20	12
Fenpropidin	0.8	2.5	106	94	8.3	8.2
Fenpropimorph	0.8	2.5	117	95	16	10
Fenpyrazamine	0.8	2.5	84	96	8.7	8.3
Fenpyroximate	1.5	5.0	102	99	17	10
Fenthion*	0.8	2.5	100	78	21	11
Fenthion Oxon Sulfoxide	0.8	2.5	105	88	8.6	17
Fenthion-Oxonsulfone	0.8	2.5	105	94	8.3	14
Fenthion-sulfone	0.8	2.5	107	100	7.0	4.5
Fenthion-sulfoxide	0.8	2.5	107	96	11	8.7
Fenthion Oxon	0.8	2.5	102	97	7.7	9.9
Fenvalerate	0.8	2.5	102	97	8.7	11
Fipronil*	0.8	2.5	88	95	21	5.3
Flonicamid	0.8	2.5	95	98	8.9	6.6
Flubendiamide	0.8	2.5	111	108	9.0	16
Flucythrinate	1.5	5.0	102	98	20	8.0

Fludioxonil	0.8	2.5	113	102	17	10
Flufenoxuron	1.5	5.0	106	96	14	13
Fluopicolide	0.8	2.5	103	103	8.6	5.1
Fluopyram	0.8	2.5	105	99	10	7.4
Fluquinconazole*	0.8	2.5	105	95	22	12
Flusilazole	0.8	2.5	107	100	9.2	8.7
Flutriafol	0.8	2.5	104	95	8.1	7.1
Fluxapyroxad	0.8	2.5	104	99	14	7.5
Folpet	1.5	5.0	89	94	18	8.0
Formetanate	0.8	2.5	103	91	8.1	5.1
Fosthiazate	0.8	2.5	103	93	7.5	8.0
Furathiocarb	1.5	5.0	103	95	20	15
Haloxyfop-P	0.8	2.5	60	74	13	1.0
Hexaconazole	0.8	2.5	109	93	19	11
Hexythiazox	0.8	2.5	113	93	20	14
Imazalil	0.8	2.5	106	89	10	10
Imidacloprid	0.8	2.5	96	99	19	7.6
Indoxacarb	0.8	2.5	98	98	7.0	7.3
Iprodione	0.8	2.5	112	81	10	19
Iprovalicarb	0.8	2.5	120	96	7.8	9.0
Isoprothiolane	0.8	2.5	86	94	13	9.9
Kresoxim-methyl	0.8	2.5	115	96	10	13
Lambda-Cyhalothrin	0.8	2.5	102	96	18	11
Linuron	0.8	2.5	103	101	6.7	9.7
Lufenuron	1.5	5.0	106	97	15	12
Malaoxon	0.8	2.5	104	91	12	10
Malathion	1.5	5.0	107	100	14	8.3
Mandipropamid	0.8	2.5	103	100	11	5.6
Mepanipyrim	0.8	2.5	104	97	15	7.0
Metaflumizone	0.8	2.5	100	99	15	5.2
Metalaxyl	0.8	2.5	105	97	8.1	9.8
Methacrifos	0.8	2.5	100	100	11	8.5
Methamidophos	0.8	2.5	95	79	18	6.4
Methidathion	0.8	2.5	96	96	14	8.3
Methiocarb	0.8	2.5	111	90	16	11
Methiocarb Sulfoxide	0.8	2.5	100	86	15	18
Methiocarb-Sulfone	0.8	2.5	92	83	4.3	2.9
Methomyl*	1.5	5.0	92	88	20	22
Methoxyfenozide	0.8	2.5	103	100	7.9	7.2
Metrafenone	0.8	2.5	102	99	20	9.6
Monocrotophos	0.8	2.5	102	86	8.5	15
Myclobutanil	0.8	2.5	77	98	7.7	3.6
Omethoate	0.8	2.5	104	90	5.2	9.8
Oxadixyl	0.8	2.5	111	91	9.2	14
Oxamyl	0.8	2.5	112	99	2.2	1.2
Oxydemeton-methyl	0.8	2.5	101	88	11	16
Paclobutrazol	0.8	2.5	100	97	3.3	1.8
Paraoxon-methyl	0.8	2.5	104	93	7.8	13
Parathion	1.5	5.0	102	94	20	7.4
Parathion-methyl	0.8	2.5	93	90	20	18
Penconazole	0.8	2.5	101	99	15	10

Pencycuron	1.5	5.0	96	94	19	12
Pencycuron PB Amine	0.8	2.5	82	96	16	7.8
Pendimethalin	1.5	5.0	97	94	19	9.0
Permethrin	0.8	2.5	111	91	15	11
Phenthoate	0.8	2.5	94	79	8.7	8.2
Phosalone	0.8	2.5	97	101	14	11
Phosmet	0.8	2.5	100	94	7.2	4.7
Pirimicarb	0.8	2.5	106	86	12	19
Pirimiphos-methyl	0.8	2.5	98	92	19	5.3
Prochloraz	0.8	2.5	101	100	17	9.7
Procymidone	0.8	2.5	107	94	16	11
Profenofos*	1.5	5.0	109	100	17	22
Propamocarb	0.8	2.5	95	88	5.4	5.8
Propargite	1.5	5.0	106	95	4.4	12
Propiconazole	0.8	2.5	109	101	20	6.6
Propyzamide	0.8	2.5	101	100	11	7.7
Proquinazid	1.5	5.0	108	94	20	12
Prosulfocarb	1.5	5.0	100	97	19	8.3
Prothioconazole Desthiometabolite	0.8	2.5	103	73	10	9.5
Prothiofos	1.5	5.0	108	92	19	9.9
Pymetrozine	0.8	2.5	67	62	9.9	9.7
Pyraclostrobin	0.8	2.5	103	98	15	7.3
Pyrazophos	0.8	2.5	103	101	13	5.0
Pyridaben	1.5	5.0	111	99	20	12
Pyridalyl	1.5	5.0	113	99	19	6.9
Pyrimethanil	0.8	2.5	107	94	11	8.2
Pyriproxyfen*	1.5	5.0	106	97	20	24
Quinoxyfen	1.5	5.0	103	94	20	17
Spinetoram J	1.5	5.0	107	90	13	13
Spinetoram L*	1.5	5.0	114	113	20	27
Spinosyn A	1.5	5.0	95	86	19	13
Spinosyn D	1.5	5.0	111	91	13	15
Spirodiclofen	1.5	5.0	108	96	20	14
Spiromesifen*	0.8	2.5	85	101	24	14
Spirotetramat	0.8	2.5	95	89	6.4	6.4
Spirotetramat-enol	0.8	2.5	118	104	6.3	9.2
Spiroxamine	1.5	5.0	95	103	20	19
Sulfoxaflor	0.8	2.5	95	94	18	20
tau-Fluvalinate	0.8	2.5	98	98	8.2	4.6
Tebuconazole	0.8	2.5	99	100	7.8	7.8
Tebufenozide	0.8	2.5	88	96	16	13
Tebufenpyrad	1.5	5.0	114	95	15	8.6
Teflubenzuron	1.5	5.0	98	105	19	12
Terbuthylazine	0.8	2.5	83	93	16	11
Tetraconazole	0.8	2.5	103	100	7.8	9.8
TFNA AM	0.8	2.5	95	95	11	9.0
Thiabendazole*	0.8	2.5	100	82	13	21
Thiacloprid	0.8	2.5	109	86	7.4	17
Thiamethoxam	0.8	2.5	109	98	20	9.6
Thiodicarb	0.8	2.5	79	94	3.4	5.0

Thiophanate-methyl	0.8	2.5	104	89	11	8.4
Tolclofos-methyl	0.8	2.5	100	98	20	10
Triadimefon	0.8	2.5	92	103	8.8	6.4
Triadimenol	1.5	5.0	113	120	15	18
Triazophos	0.8	2.5	104	94	7.3	6.0
Tricyclazole	0.8	2.5	104	84	12	20
Trifloxystrobin	0.8	2.5	105	97	14	10
Triflumuron	0.8	2.5	102	97	14	10
Trifluralin	0.8	2.5	93	98	12	6.9

*Analytes with one validation parameter outside the ranges reported in SANTE\11312\2021 v2 have been considered for qualitative determination only.

Table S5. LOD, LOQ, average recovery, and precision at low and high concentrations for *Alternaria* Toxins

Target Analyte	LOD (μg/kg)	LOQ (μg/kg)	Average Rec% (LOQ)	Average Rec% (LOQ x5)	RSD_r (LOQ)	RSD_r (LOQ x5)
TeA	23	75	110	91	6.0	6.5
ALT	0.5	1.5	109	108	8.9	9.9
AOH	0.5	1.5	112	96	4.3	7.4
TEN	0.5	1.5	109	89	5.2	6.4
AME	0.5	1.5	99	102	4.7	8.5

Table S6. Exposure assessment and risk characterization of *Alternaria* toxins in tomato sauce samples

Toxin - Group	Mean O (n=20) μg/kg	% left-censored data	Mean C (n=20) μg/kg	% left-censored data	Total mean (n=40) μg/kg	% left-censored data	Dietary Exposure μg/kg bw (O)	Dietary Exposure μg/kg bw (C)	Dietary Exposure μg/kg bw (T)
**All - World**									
**TeA**	88.48	15	99.42	10	93.95	12.5	0.035393	0.039767	0.037580
**TEN**	0.60	40	0.53	15	0.57	27.5	0.000239	0.000213	0.000226
**AOH**	2.68	75	4.80	95	3.74	85	0.001073	0.001918	0.001496
**AME**	1.00	50	1.23	30	1.12	40	0.000401	0.000493	0.000447
**All - European Union**									
**TeA**	88.48	15	99.42	10	93.95	12.5	0.0619	0.0696	0.0658
**TEN**	0.60	40	0.53	15	0.57	27.5	0.0004	0.0004	0.0004
**AOH**	2.68	75	4.80	95	3.74	85	0.0019	0.0034	0.0026
**AME**	1.00	50	1.23	30	1.12	40	0.0007	0.0009	0.0008
**All - Italy**									
**TeA**	88.48	15	99.42	10	93.95	12.5	0.0575	0.0646	0.0611
**TEN**	0.60	40	0.53	15	0.57	27.5	0.0004	0.0003	0.0004
**AOH**	2.68	75	4.80	95	3.74	85	0.0017	0.0031	0.0024
**AME**	1.00	50	1.23	30	1.12	40	0.0007	0.0008	0.0007
**p95 - Italy**									
**TeA**	88.48	15	99.42	10	93.95	12.5	0.1495	0.1680	0.1588
**TEN**	0.60	40	0.53	15	0.57	27.5	0.0010	0.0009	0.0010

**AOH**	2.68	75	4.80	95	3.74	85	0.0045	0.0081	0.0063
**AME**	1.00	50	1.23	30	1.12	40	0.0017	0.0021	0.0019
**Infants - Italy**									
**TeA**	88.48	15	99.42	10	93.95	12.5	0.1018	0.1143	0.1080
**TEN**	0.60	40	0.53	15	0.57	27.5	0.0007	0.0006	0.0007
**AOH**	2.68	75	4.80	95	3.74	85	0.0031	0.0055	0.0043
**AME**	1.00	50	1.23	30	1.12	40	0.0012	0.0014	0.0013
**Other children - Italy**									
**TeA**	88.48	15	99.42	10	93.95	12.5	0.1602	0.1799	0.1700
**TEN**	0.60	40	0.53	15	0.57	27.5	0.0011	0.0010	0.0010
**AOH**	2.68	75	4.80	95	3.74	85	0.0049	0.0087	0.0068
**AME**	1.00	50	1.23	30	1.12	40	0.0018	0.0022	0.0020
**Toddlers - Italy**									
**TeA**	88.48	15	99.42	10	93.95	12.5	0.2460	0.2764	0.2612
**TEN**	0.60	40	0.53	15	0.57	27.5	0.0017	0.0015	0.0016
**AOH**	2.68	75	4.80	95	3.74	85	0.0075	0.0133	0.0104
**AME**	1.00	50	1.23	30	1.12	40	0.0028	0.0034	0.0031
**Adolescents - Italy**									
**TeA**	88.48	15	99.42	10	93.95	12.5	0.0867	0.0974	0.0921
**TEN**	0.60	40	0.53	15	0.57	27.5	0.0006	0.0005	0.0006
**AOH**	2.68	75	4.80	95	3.74	85	0.0026	0.0047	0.0037
**AME**	1.00	50	1.23	30	1.12	40	0.0010	0.0012	0.0011
**Adults - Italy**									
**TeA**	88.48	15	99.42	10	93.95	12.5	0.0540	0.0606	0.0573
**TEN**	0.60	40	0.53	15	0.57	27.5	0.0004	0.0003	0.0003
**AOH**	2.68	75	4.80	95	3.74	85	0.0016	0.0029	0.0023
**AME**	1.00	50	1.23	30	1.12	40	0.0006	0.0008	0.0007
**Elderly - Italy**									
**TeA**	88.48	15	99.42	10	93.95	12.5	0.0628	0.0706	0.0667
**TEN**	0.60	40	0.53	15	0.57	27.5	0.0004	0.0004	0.0004
**AOH**	2.68	75	4.80	95	3.74	85	0.0019	0.0034	0.0027
**AME**	1.00	50	1.23	30	1.12	40	0.0007	0.0009	0.0008

O = organic
C = conventional
T = total
Indicative level (Recommendation (EU) 2022/553): TEA: 500 μg/kg; AOH 10 μg/kg; AME: 5 μg/kg
TTC (TeA and TEN) = 1.5 μg/kg bw day
TTC (AOH and AME) = 0.0025 μg/kg bw day

Table S7. Consumption data tomato sauces/puree

Consumption data FAOSTAT (2010 - 2021)		
**Population class**	Mean consumption estimated value tomato sauces/puree (processed not concentrated) g/day	Mean consumption estimated value tomato sauces/puree (processed not concentrated) g/kg bw per day
**All - World**	24.16	0.40
**All - European Union**	42.28	0.70
**All - Italy**	38.76	0.65
**Consumption data INRAN SCAI (Italy - 2006)**		
**Population class**	Mean consumption (g/day) Processed vegetables	Mean consumption (g/kg bw per day) Processed vegetables
**All**	47.10	0.79
**Median**	39.40	0.66
**P-95**	101.40	1.69
**P-99**	145.50	2.43
**FoodEX-EFSA (Italy - 2018)**		
**Population class (Italy)**	Mean consumption (g/day) tomato puree	Mean consumption (g/kg bw per day) tomato puree
**Infants**	10.80	1.15
**Other children**	35.92	1.81
**Toddlers**	36.67	2.78
**Adolescents**	43.33	0.98
**Adults**	39.17	0.61
**Elderly**	48.33	0.71
